# Identification of the mulberry genes involved in ethylene biosynthesis and signaling pathways and the expression of *MaERF*-*B2*-1 and *MaERF*-*B2*-*2* in the response to flooding stress

**DOI:** 10.1007/s10142-014-0403-2

**Published:** 2014-09-18

**Authors:** Jingzhe Shang, Penghua Song, Bi Ma, Xiwu Qi, Qiwei Zeng, Zhonghuai Xiang, Ningjia He

**Affiliations:** State Key Laboratory of Silkworm Genome Biology, Southwest University, Beibei, Chongqing, 400715 China

**Keywords:** Ethylene, Plant hormone, Mulberry, Gene expression

## Abstract

The phytohormone ethylene is essential to plant growth and development. It plays crucial roles in responses to biotic and abiotic stress. The mulberry tree is an important crop plant in countries in which people rear silkworms for silk production. The availability of the mulberry genome has made it possible to identify mulberry genes involved in ethylene biosynthesis and signal pathways. A total of 145 mulberry genes were identified by both homology-based and hidden Markov model (HMM) search, including 29 genes associated with ethylene biosynthesis and 116 genes in the AP2/ERF family. Studies on gene structure have provided a genetic basis for understanding the functions of these genes. The differences in gene expression were also observed in different tissues. The expression of two mulberry genes in the AP2/ERF family, *MaERF*-*B2*-*1* and *MaERF*-*B2*-2, was found to be associated with the response to flooding stress.

**Electronic supplementary material:**

The online version of this article (doi:10.1007/s10142-014-0403-2) contains supplementary material, which is available to authorized users.

## Introduction

Ethylene plays important roles in various aspects of plant growth and development including fruit ripening, germination, flowering, sex determination, leaf senescence, root nodulation, and responses to biotic and abiotic stress (Johnson and Ecker 1998; Lin et al. 2009). As early as 1910, ethylene was used to accelerate the ripening of bananas (Abeles et al. 1992). In *Arabidopsis*, ethylene signaling has been found to be important to flooding and salt response (Zhang et al. 2011; Voesenek and Blom 1989). Recent work has indicated that ethylene promotes hypocotyl elongation in light and suppresses it in darkness (Zhong et al. 2012).

Ethylene biosynthesis and signaling networks are well understood in plants. Biosynthesis of ethylene occurs in three steps. First, the amino acid methionine is converted to S-adenosylmethionine (AdoMet) by S-adenosylmethionine synthetase (Yang and Hoffman 1984). AdoMet is then converted to 1-aminocyclopropane-1-carboxylic acid (ACC) and 5′-deoxy-5′methylthioadenosine (MTA) by 1-aminocyclopropane-1-carboxylase synthase (ACS) (Adams and Yang 1979). The formation of the ACC is the rate-limiting step in ethylene biosynthesis, and it requires pyrodoxal-5′-phosphate (PLP) as a cofactor. When a large amount of ethylene is produced, MTA is recycled to methionine through the Yang cycle to supplement the methionine pool (Miyazaki and Yang 1987). In the third step, ACC is transformed into ethylene, CO_2_, and cyanide by 1-aminocyclopropane-1-carboxylase oxidase (ACO). The cyanide is detoxified into β-cyanoalanine by β-cyanoalanine synthase (Blumenthal et al. 1968). Ethylene can be detected by a family of membrane-localized receptors. A copper cofactor is necessary for the transduction of this signal (Rodrıguez et al. 1999). When ethylene is present, the conformation of the compound of ethylene receptor and constitutive triple response 1 (*CTR1*) changes and the suppression ends (Gao et al. 2003). Ethylene-insensitive 2 (*EIN2*) is then activated, resulting in the activation of *EIN3* transcription factors (Alonso et al. 1999). Proteins EIN3 and EIN3-like 1 (EIL1) are specifically bound to the promoter of ethylene response factor 1 (ERF1) in AP2/ERF family, and ERF1 plays important roles in the transcriptional regulation of a variety of biological processes associated with growth and development and with biotic and abiotic stress responses. ERF1 binds to the promoters that contain the GCC box. These promoters are present in many inducible ethylene genes (Fujimoto et al. 2000). The related apetala 2.2 gene from *Arabidopsis* (*RAP2.2*, *At3g14230*) has an ERF-bearing AP2 domain (Hinz et al. 2010). Overexpression of *RAP2.2* has been shown to improve plant survival under hypoxia stress conditions. The ERF-associated amphiphilic repression (EAR) motif is a repression domain in the C-terminal region of repressor-type ERF proteins. These genes are implicated in developmental, hormonal and stress signaling pathways and can be negatively regulated through the EAR motif (Ohta et al. 2001; Kagale et al. 2010).

Mulberry trees are deciduous and woody. They are widely used to produce crops for the sericulture industry. The domesticated silkworm, *Bombyx mori*, feeds only on mulberry leaves. In this way, the silk industry depends on the quality and quantity of available mulberry leaves. Mulberry fruits are also used to make jam, juice, and wine. Ethylene has been shown to affect seasonal leaf abscission and fruit ripening (Adams-Phillips et al. 2004). The genes involved in ethylene signaling and biosynthesis are important targets for the improvement of leaf and fruit yield. In China’s Three Gorges Reservoir Region, the most serious type of environmental stress limiting mulberry crop productivity is water-logging. ERFs have been reported to play important roles in the regulation of biotic and abiotic stress (Dietz et al. 2010). One ERF subfamily, the VIIERFs, responds to hypoxia stress and promotes the expression of hypoxia-related genes (Hinz et al. 2010). In the present study, we found the expression patterns of two mulberry genes in the AP2/ERF family, *MaERF*-*B2*-*1* and *MaERF*-*B2*-*2*, changed in association with flooding treatment.

## Materials and methods

### Database searches and sequence analysis

The mulberry genome database was downloaded from MorusDB (http://morus.swu.edu.cn/morusdb/). The *Arabidopsis* genome database was obtained from TAIR (http://www.arabidopsis.org/). The majority of amino acid sequences of the *Arabidopsis thaliana* genes involved in the ethylene biosynthesis and signaling pathways were retrieved from Uniprot (http://www.uniprot.org/). The *A. thaliana* AP2/ERF genes were downloaded from the DATF website (http://datf.cbi.pku.edu.cn/index.php). All of the *A. thaliana* sequences were used as templates in a search of the mulberry genome using BLASTP. Hidden Markov model (HMM) was used to identify motifs of mulberry proteins. The presence of AP2 domains in the mulberry sequences identified in this way was determined by (http://smart.embl-heidelberg.de/) (SMART) analyses (Letunic et al. 2012).

### Sequence alignments and construction of the phylogenetic tree

Multiple alignment analysis was performed with ClustalX and subsequently adjusted manually by Genedoc (Larkin et al. 2007). A phylogenetic tree of AP2/ERF was constructed using maximum-likelihood method in PhyML 3.0 using a Perl script. Web tools at the Gene Structure Display Server (http://gsds.cbi.pku.edu.cn/help.php) were used to draw the structures of the genes (Guo et al. 2007).

### Analysis of the expression of the genes involved in ethylene biosynthesis and signal transduction

The reads per kilobase of exon model per million mapped reads (RPKM) was used to analyze the expression levels of all predicted mulberry genes using RNA sequencing data (Mortazavi et al. 2008). The clustering analyses were carried out using Gene Cluster 3.0. The data were adjusted by log transformation and the mean center method. Hierarchical clustering (HCL) with average linkage was set to calculate K-medians with five clusters. Heat maps illustrating the gene expression data were generated using Java TreeView.

### RNA extractions and quantitative RT-PCR

Total RNA was isolated from mulberry roots, barks, leaves, male flowers, female flowers, and fruits using RNAiso Plus (Takara, Japan) according to the manufacturer’s instructions. The quality and concentration of RNA samples were measured using a ND-1000 UV spectrophometer (Nanodrop Technologies, USA). Reverse transcription was performed following the manufacturer’s instructions (Takara, Japan). Primers were designed using Primer Premier 5 software (http://www.primer-e.com/). Polymerase chain reactions were performed in a 96-well plate with a StepOne Plus System apparatus, amplified with SYBR® Green II (Takara, Japan) according to the manufacturer’s instructions. Cycling conditions were as follows: 90 °C for 30 s; 40 cycles of 95 °C for 5 s and 60 °C for 30 s. Amplification specificity was verified by a dissociation curve. To compare data from different PCR runs, cycle threshold (*C*_T_) values for samples were normalized to that of the mulberry ribosomal protein gene (*Morus024083*). The cDNA quantity was calculated using ${2}^{-\varDelta {C}_{\mathrm{T}}}$; here, Δ*C*_T_ is the difference in the number of cycles between tests and controls. The change in gene expression after flooding treatment was measured using ${2}^{-\varDelta \varDelta {C}_{\mathrm{T}}}$, where ΔΔC_*T*_ = (ΔC_*T*_ sample − ΔC_*T*_ control). Gene-specific primers used for the real-time RT-PCR are listed in Supplementary Table 2. All results are representative of three independent experiments.

### Prediction of *cis*-acting elements

To identify *cis*-regulatory elements, upstream regions within about 2 kb of the transcription start sites of three genes, *MnERF*-*B1*, *MnERF*-*B*2, and *MnERF*-*B3*, were analyzed. All promoter sequences were placed in the PlantCare database (http://bioinformatics.psb.ugent.be/webtools/plantcare/html), which has 435 different plant transcription binding sites. The search results are divided into four categories including stress response, hormone response, light response, and others (Lescot et al. 2002).

### Plant growth and flooding treatment

Mulberry species, Husang-32 (*Morus alba* L. var. multicaulis), maintained at the Mulberry Germplasm Nursery of Southwest University (China), was used in the present study. Husang-32 seedlings were grown in plastic pots filled with soil, vermiculite, and perlite mixture (6:1:1) in incubators (25 °C; 16 h day, 8 h night; 75 % relative humidity). After being incubated for 2 months, the seedlings with 15-cm length were subjected to flooding treatment. Plants in the flooding-treated group were submerged 2–3 cm under the surface of the water. The roots and leaves from both treated and control groups of Husang-32 were collected after the treatments lasting 1 h, 3 h, 5 h, 7 h, 1 day, 3 days, 5 days, and 7 days. Plant materials were frozen in liquid N_2_, and stored at −80 °C until further use for total RNA isolation.

## Results

### Identification of genes involved in the mulberry ethylene biosynthesis and signaling pathways

**Fig. 1:**
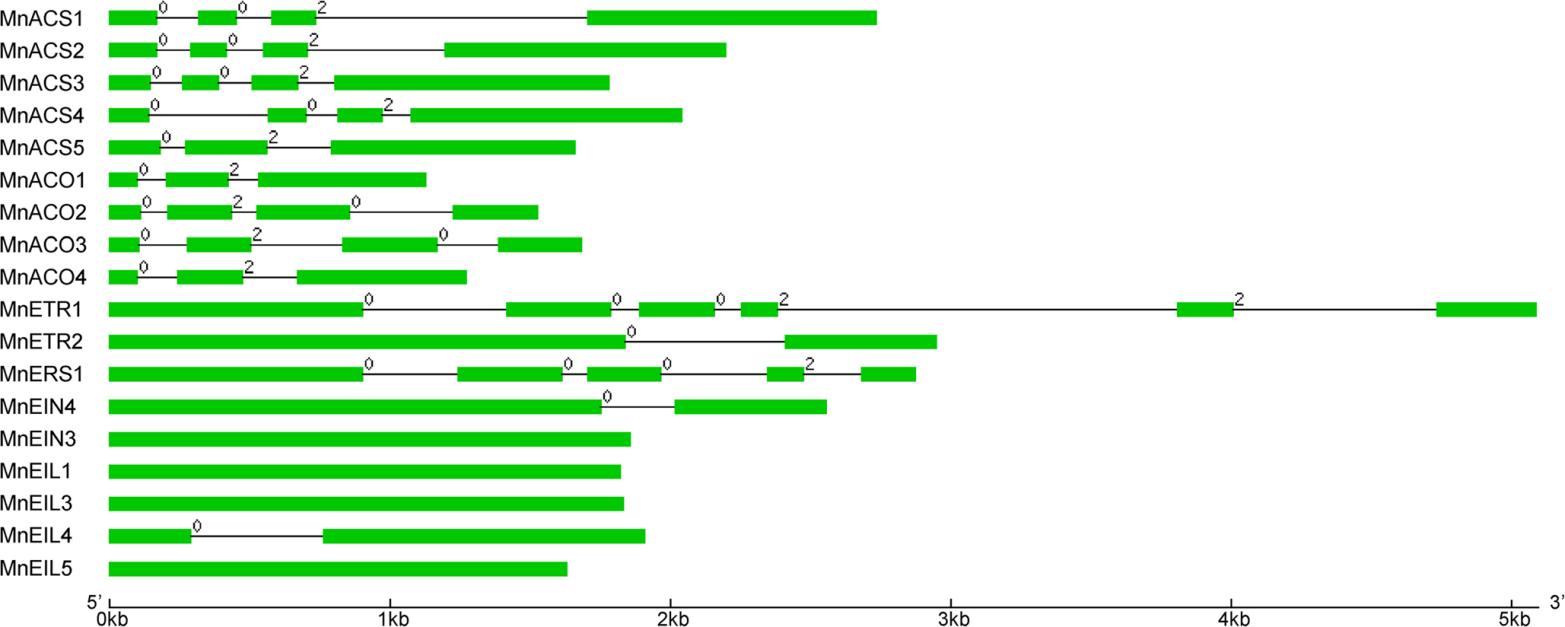
Exon/intron structures of *ACS*, *ACO*, *ETR*, and *EIN3* genes in mulberry plants. Gene structures were plotted using the GSDS server. The *intron phases* indicate the position of the intron within a codon. The *scale bar* indicates 1 kb

In total, 145 genes were predicted to belong to the ethylene biosynthesis and signaling pathways. Of these, 29 genes were found to be involved in ethylene biosynthesis and signal transduction (Table 1). The AP2/ERF family contains 116 genes (Supplemental Table 1). The *MnACS* family contains 7 genes, of which *MnACS10* and *12* are transaminases. Four of the remaining five *MnACS* genes each had four exons. *MnACS5* only had three exons (Fig. 1). Sequence alignment indicated that each MnACS protein contained seven conserved boxes also found in ACS proteins from other plant species. The conserved glutamate (E) residue in box 1, which determines substrate specificity, is present in all the members of the ACS gene family. Except for MnACS5, the MnACS proteins all shared a Ser residue in the C-terminal, which produced a shorter C-terminus than other ACS isozymes (Supplemental Figure 1). ACO is a member of the Fe II-dependent family of oxidases. They require ascorbate as a substrate and Fe (II) as a cofactor to exert enzymatic activity. Four *MnACOs* were identified in the mulberry genome. *MnACO1* and *MnACO4* have three exons, and *MnACO2*/*3* has four exons (Fig. 1). The motifs for binding the cofactor (H-X-D-X-H) and the cosubstrate (R-X-S) are also conserved in *MnACOs*. In the C-terminus, E-R-E is essential to enzyme activity, particularly R, which may be involved in the mechanism of CO_2_ activation (Supplementary Figure 2).

**Table 1:** Genes involved in ethylene biosynthesis and signaling pathways in the *M. notabilis* genome

Genes	Accession no.	Protein size	CDS length	Gene length	Exons	Scaffold. no. (strand)	Start codon	Stop codon
MnSAM1	Morus003267	393	1182	1182	1	1843(+)	66,189	67,370
MnSAM2	Morus013867	393	1182	1182	1	498(+)	76,865	78,046
MnSAM3	Morus025140	393	1182	1182	1	172(+)	571,546	572,727
MnSAM4	Morus022555	390	1173	1173	1	124(+)	13,770	14,942
MnACS1	Morus012919	496	1491	2732	4	298(+)	185,423	188,154
MnACS2	Morus024218	486	1461	2196	4	297(+)	4647	6842
MnACS3	Morus007775	471	1416	1782	4	300(−)	229,933	231,714
MnACS4	Morus007092	467	1404	2044	4	991(+)	117,590	119,633
MnACS5	Morus027243	446	1341	1660	3	329(−)	186,543	188,202
MnACS10^a^	Morus023174	557	1674	3428	5	87(+)	408,302	411,729
MnACS12^a^	Morus012967	504	1515	1791	4	399(+)	330,934	332,724
MnACO1	Morus027261	306	921	1127	3	329(+)	408,750	409,876
MnACO2	Morus014137	322	969	1526	4	1292(−)	16,575	18,100
MnACO3	Morus004820	319	960	1682	4	528(−)	23,279	24,960
MnACO4	Morus013401	310	933	1272	3	1379(+)	391,862	393,133
MnETR1	Morus018344	738	2217	5082	6	521(−)	266,232	271,313
MnETR2	Morus008145	793	2382	2949	2	606(−)	247,113	250,061
MnERS1	Morus007485	617	1854	2872	5	1526(+)	209,537	212,408
MnEIN4	Morus024538	764	2295	2558	2	205(−)	594,177	596,734
MnCTR1	Morus003569	861	2586	6563	17	548(+)	106,465	113,027
MnCTR2	Morus016797	666	2001	6117	12	112(−)	213,548	219,664
MnEIN2	Morus024376	1306	3921	5614	7	342(−)	402,131	407,744
MnEBF1	Morus000805	697	2094	2590	2	1037(+)	4387	6976
MnEBF2	Morus002248	642	1929	2397	2	1513(−)	59,843	62,239
MnEIN3	Morus002490	617	1854	1854	1	3287(−)^b^	31,544	33,397
MnEIL1	Morus002491	607	1824	1824	1	3287(−)^b^	50,362	52,185
MnEIL3	Morus007978	611	1836	1836	1	18(−)	151,180	153,015
MnEIL4	Morus016592	478	1437	1910	2	841(+)^b^	82,684	84,593
MnEIL5	Morus016593	543	1632	1632	1	841(+)^b^	86,287	87,918

*SAM* S-adenosylmethionine synthase, *ACS* 1-aminocyclopropane-1-carboxylate synthase, *ACO* 1-aminocyclopropane-1-carboxylate oxidase, *ETR* ethylene receptor, *CTR1* constitutive triple response-1, *EIN2* ethylene-insensitive 2, *EIN3* ethylene-insensitive 3, *EBF* EIN3-binding F-box protein, *ERF* ethylene-responsive element binding factor.

^a^The transaminases in ACS family

^b^The genes located at the same scaffold

Ethylene signal transduction is important to ethylene synthesis. Many genes take part in this process. Ethylene receptors (ETRs) form a family of membrane-localized receptors. There are four *MnETRs* in the mulberry genome. The N-terminal of *MnETRs* includes three highly conserved transmembrane regions. This region has been demonstrated to be essential to the ethylene binding activity of *MnETRs*. The C-terminal region of all *MnETRs* except *MnERS1*, which has a truncated C-terminal, contains a receiver domain. In addition, the N-terminal regions of *MnETR2* and *MnEIN4* are much longer than those of other receptors. SMART prediction revealed that they contain four transmembrane regions (Supplementary Figure 3). EIN3 and EIN3-like proteins are essential to the ethylene signal pathway. They are positive regulators, and they bind to the promoter regions of the downstream genes to regulate their expression. Five *EIN3s* were found here in the mulberry genome. They all have a conserved amino-terminal acidic domain (AD), pro-rich region (PR), and five small basic domains (BDI-V). The Gln-rich and Asn-rich regions, found in mung bean plants, are only conserved in *MnEIN3* and *MnEIL1* (Supplementary Figure 4). *MnEIN3*, *MnEIL1*, *MnEIL4*, and *MnEIL5* were all located on the same scaffold in the mulberry genome (Table 1).

### Expression of mulberry ethylene biosynthesis and signal pathway genes

**Fig. 2:**
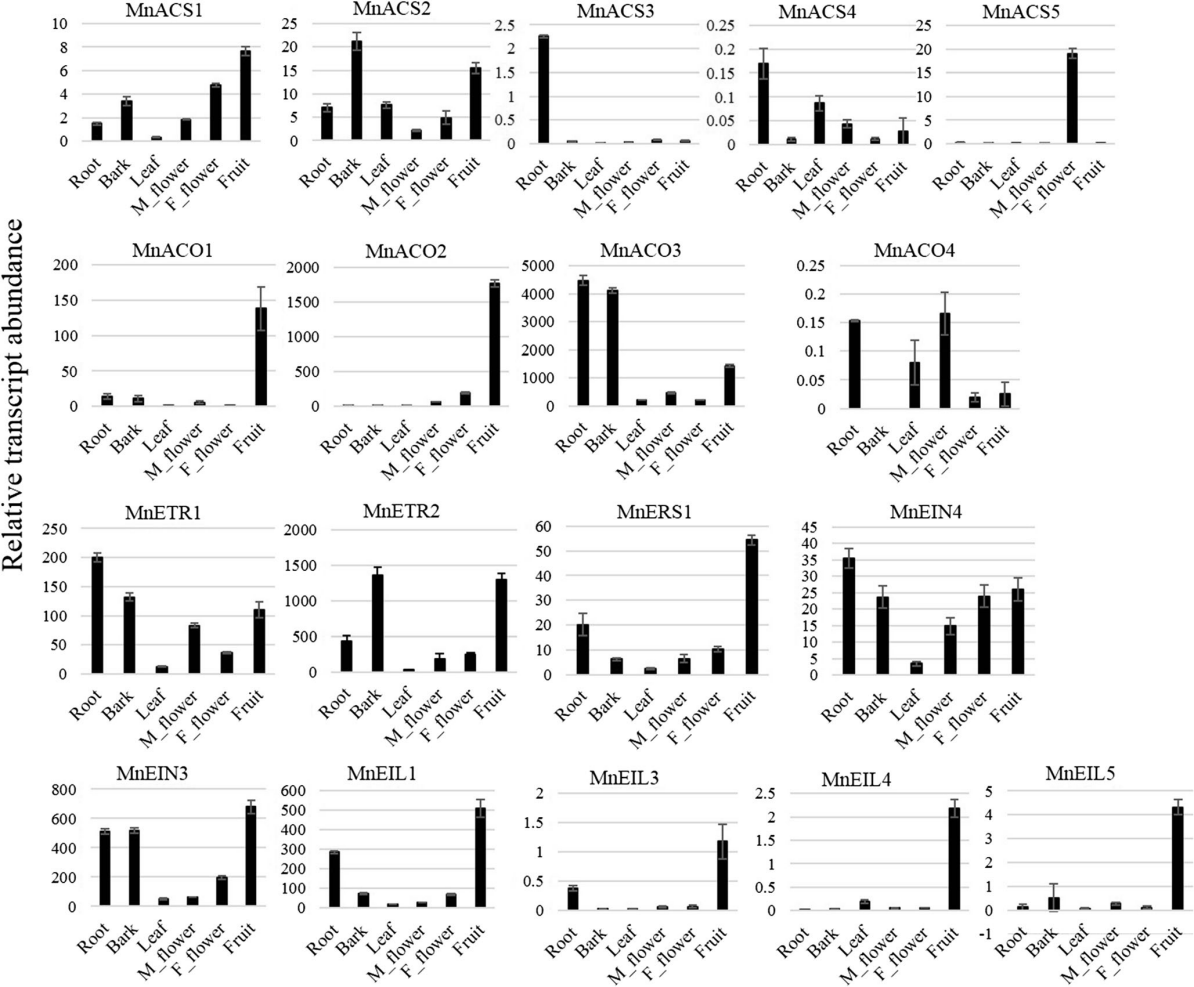
Expression patterns of 18 genes involved in the ethylene biosynthesis and signal transduction pathways. Six tissues were used. Relative levels of gene expression by qRT-PCR were normalized against the mulberry ribosomal protein gene *Morus024083*. Data are represented as mean ± standard error of three replicates

Genes whose expression was detected by qPCR are listed in Fig. 2. Results showed that the genes in these four families have diverse expression profiles across different tissues. *MnACS2*, *MnACO2*, *MnACO3*, *MnETR2*, *MnEIN3*, and *MnEIL1* have higher levels of expression. On the contrary, the expression of *MnACS3*, *MnACS4*, *MnACO4*, *MnERS1*, *MnEIN4*, *MnEIL3*, and *MnEIL4* was relatively repressed. In addition, *MnACS5* exhibited tissue-specific expression in female flowers. Among ACO genes, *MnACO1* and *MnACO2* exhibited tissue-biased expression in fruit, although the expression levels of *MnACO1* were only about one tenth of those of *MnACO2. MnEIN3* and *MnEIL1* showed more expression than the rest of the EIN3 family in roots and fruits.

### Phylogenetic and structural analysis of the AP2/ERF transcription factor family

In the present study, 116 AP2/ERF transcription factors were identified in the mulberry genome. According to the number of AP2 domains and their structural features, these 116 proteins can be divided into five subfamilies. There are 58 genes in the ERF subfamily, 33 genes in the DREB subfamily, 21 genes in the AP2 subfamily, 3 genes in the RAV subfamily, and 1 gene in the Soloist subfamily. A phylogenetic tree was constructed using the amino acid sequences of AP2/ERF transcription factors of *Morus* and *Arabidopsis*. This tree was separated into 15 groups. Groups I–IV represent the DREB subfamily, and V–X represent the ERF subfamily (Supplementary Figure 5). All groups except for the V group have lower numbers of genes in mulberry tree than those in Arabidopsis. There are 11 genes in the V group of mulberry and 5 in the V group of *Arabidopsis*.

The pattern of exon/intron splicing usually provides information useful to understanding of the emergence and evolution of a gene family. Structural analyses of genes indicated that all genes in the AP2 subfamily had different numbers of introns ranging from zero to twelve. However, 28 of the 33 genes in the DREB subfamily, 44 of the 58 genes in the ERF subfamily, and 2 of the 3 genes in the RAV subfamily had no introns. Results showed that most genes in the AP2 family shared similar patterns of exon/intron splicing (Supplementary Figure 6).

### Genes in the AP2/ERF family containing the EAR motif

**Fig. 3:**
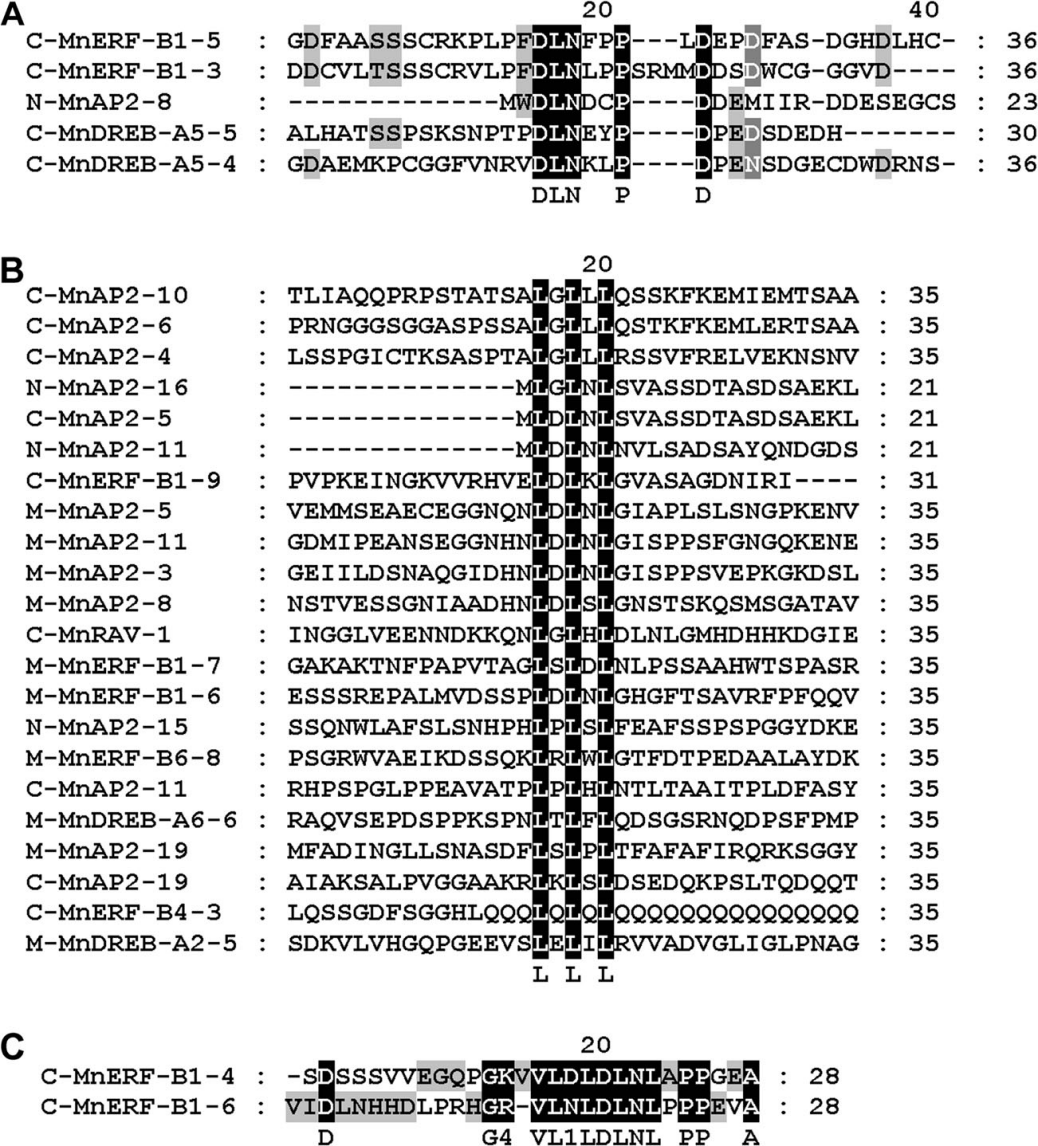
EAR motif-like sequences of AP2/ERF in mulberry plants. **a** The genes contain DLNXXP motif. **b** The genes contain LXLXL motif. **c** The genes contain LDLNLXPP motif. Multiple sequence alignments were performed using ClustalX. The letters *C*, *M*, and *N* prior to protein names indicate location in the C-terminal, middle of the sequence, and N-terminal

The ERF-associated amphiphilic repression (EAR) motif (DLNxxP or LXLXL) has been reported to be a repression domain in repressor-type ERF proteins. These proteins negatively regulate genes involved in developmental, hormonal, and stress signaling pathways. There are three types of EAR motifs in mulberry plants, DLNXXP, LXLXL, and LDLNLXPP. These three motifs have been found in ERF, DREB, AP2, and RAV subfamily (Fig. 3). All of MnERF proteins bearing EAR motif belong to MnERF-B1 group except MnERF-B4-3 and MnERF-B6-8. Only four genes in MnDREB subfamily contain EAR motif, and the DLNXXP motif has been identified in *MnDREB*-*A5*-*4* and *MnDREB*-*A5*-*5*. Sequence analyses revealed the 19 genes that bear LXLXL motif found in mulberry proteins.

### Expression of *MnERF* genes

**Fig. 4:**
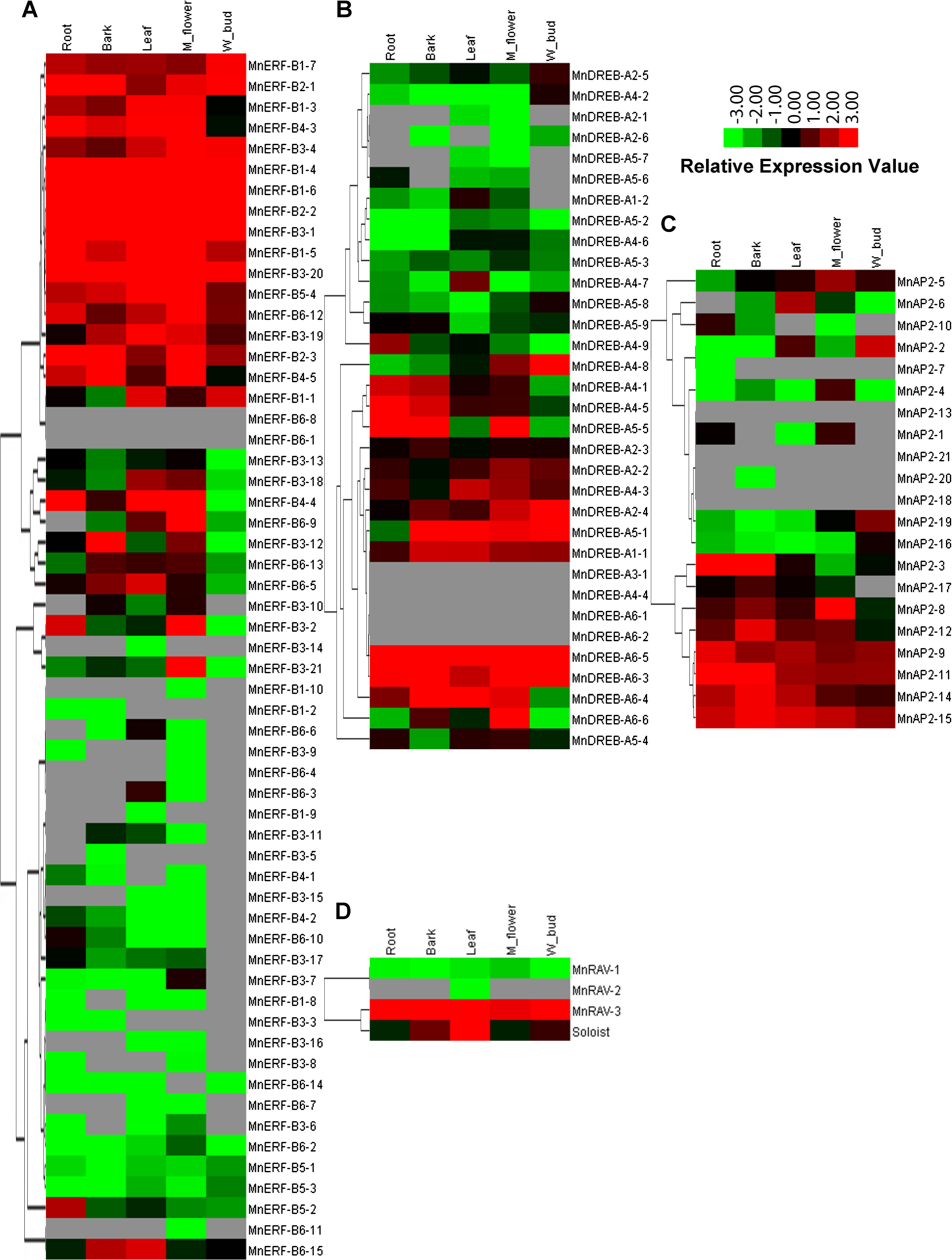
Heat maps of hierarchical clustering of mulberry genes in the AP2/ERF family. **a** ERF subfamily; **b** DREB subfamily; **c** AP2 subfamily; and **d** RAV and Soloist subfamilies. The clustering analyses were carried out using Gene Cluster 3.0. The data were adjusted by log transformation and the mean center method. Hierarchical clustering (HCL) with average linkage was set to calculate K-medians with five clusters. Heat maps illustrating the gene expression data were generated using Java TreeView

To investigate the levels of expression of AP2/ERF genes in different tissues, RPKM data of these genes were analyzed in roots, barks, leaves, flowers, and buds. Heat maps were constructed based on the RPKM data. Sixteen *MnERF* genes, including five genes in MnERF-B1, four in MnERF-B3, three in MnERF-B2, two in MnERF-B4, and one each in MnERF-B5 and MnERF-B6, had relatively high levels of expression in five tissues (Fig. 4a). This was also true of 8 of the 33 genes in the DREB subfamily, 6 of the 21 genes in the AP2 subfamily, and 1 of the 3 genes in the RAV subfamily. In addition, several genes were also found to be expressed in a tissue-biased manner. For example, *MnERF*-*B3*-*21* was expressed solely in male flowers (Fig. 4a). *MnDREB*-*A4*-*7* was more abundantly expressed in leaves than in other tissues (Fig. 4b). *MnAP2*-*5* had high levels of expression in male flower (Fig. 4c). However, the expression of nine genes (*MnERF*-*B6*-*1*/*8*, *MnDREB*-*A3*-*1*, *MnDREB*-*A4*-*4*, *MnDREB*-*A6*-*1*/*2*, *MnAP2*-*13*/*18*/*21*) was not detectable in any tissue.

### Response of *MaERF*-*B2*-*1* and *MaERF*-*B2*-*2* to flooding stress

**Fig. 5:**
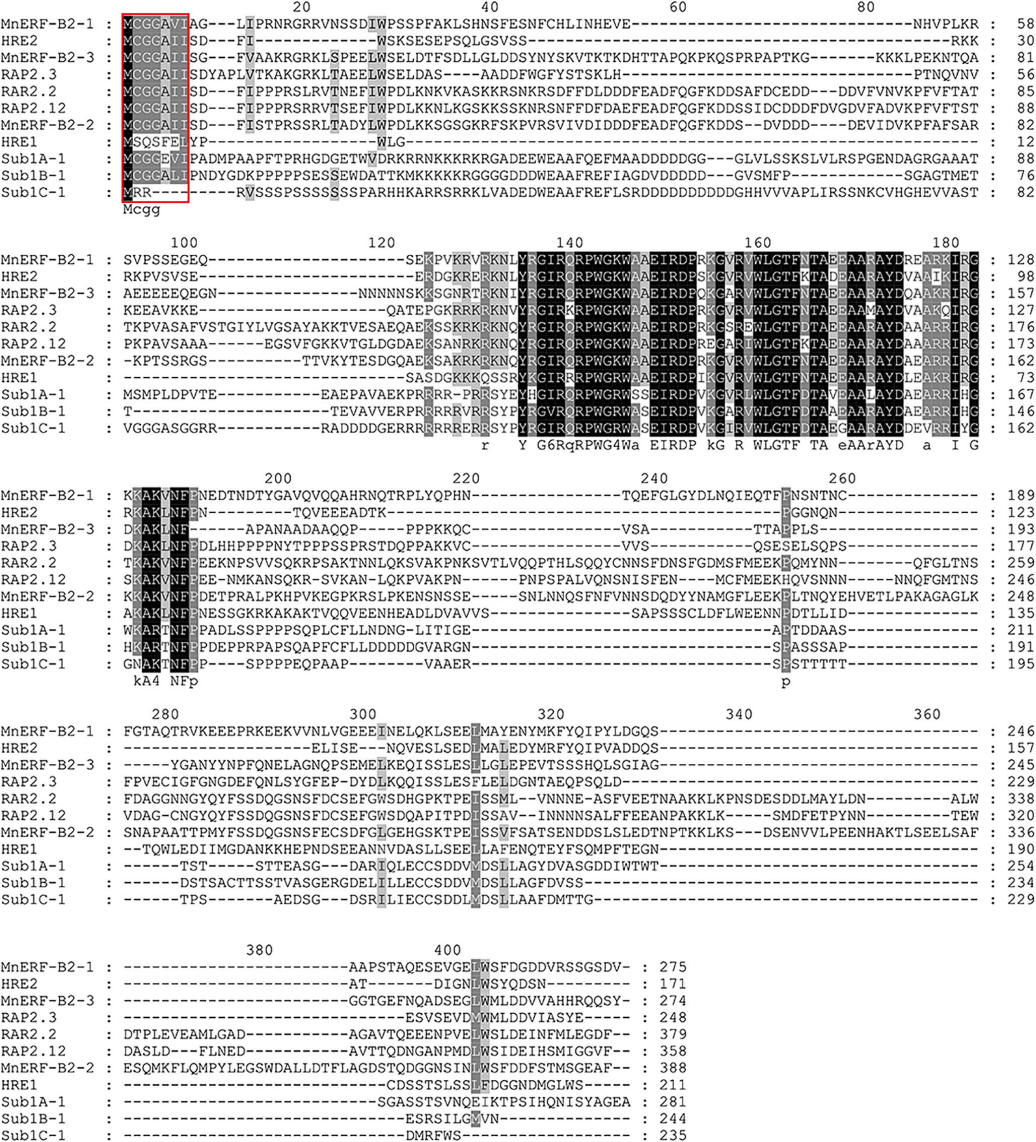
Alignment of amino acid sequences of ERF VII subfamily genes from mulberry, Arabidopsis and rice using the ClustalX program. HRE1/2, RAP2.2, RAP2.3, and RAP2.12 belong to VII group of *Arabidopsis*. Sub1A-1, Sub1B-1, and Sub1C-1 belong to *Oryza sativa*

**Fig. 6:**
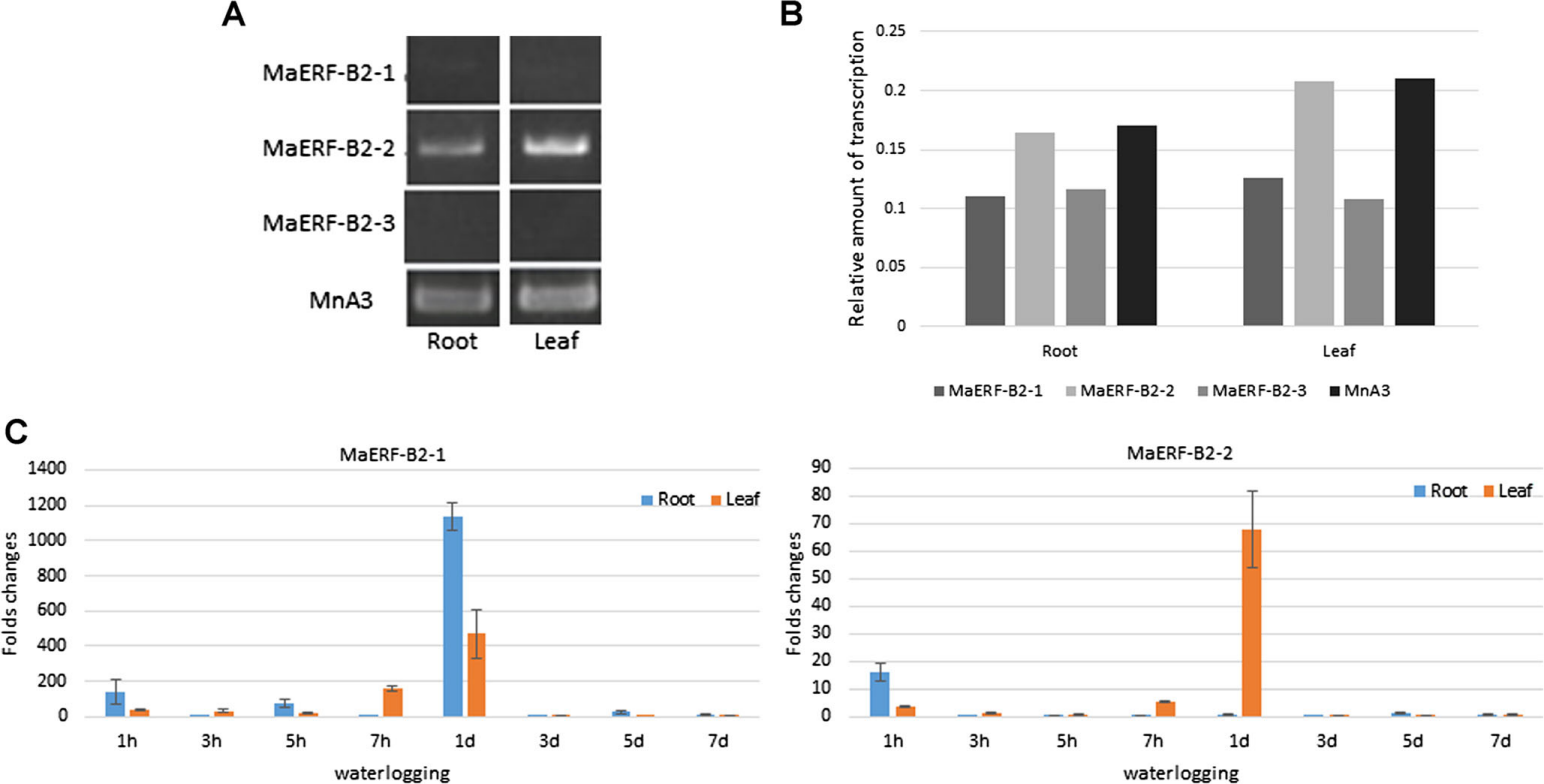
Expression analyses of *MaERF*-*B2*-*1*/*2*/*3* genes. Expression of *MaERF*-*B2*-*1*, *MaERF*-*B2*-*2*, and *MaERF*-*B2*-*3* in roots and leaves (**a**, **b**). The qPCR data of *MaERF*-*B2*-*1* and *MaERF*-*B2*-*2* in roots and leaves after flooding treatment (**c**). Fold inductions are calculated by dividing the value of treatment with that of control in corresponding time point. Data are represented as mean ± standard error of three replicates

Genes in MnERF-B subfamily were expressed at relatively higher levels in different tissues. All members of this subfamily have a conserved N-terminal motif MCGGAV/II, which is considered to be involved in the regulation of low-oxygen response in plants (Fig. 5). Analyses of the promoter regions of *MnERF*-*B2*-*1*, *MnERF*-*B2*-*2*, and *MnERF*-*B2*-*3* showed that they contain consensus sequences of transcription factor binding sites. As shown in Table 2, four catalogues of putative regulatory elements (stress response, hormone response, light response, and others) were detected in the flanking regions of three genes. GARE, CE3, ABRE, TCA, and TGACG-motifs are involved in the signaling of mulberry hormones. Typical heat shock element (HSE) and low temperature stress response element (LTR) were also observed. It is noteworthy that ARE and GC motifs, which are known to be responsive to the hypoxemia and anaerobic conditions, were detected in the promoters of *MnERF*-*B2*-1 and *MnERF*-*B2*-*3*. In this context, two of these three genes from Husang-32, *MaERF*-*B2*-*1* and *MaERF*-*B2*-*2*, were cloned, sequenced, and their expression patterns were further investigated. Mulberry cultivar Husang-32 is widely planted in the Three Gorges Reservoir area. As shown in Fig. 6a, *MaERF*-*B2*-*2* has higher expression than *MaERF*-*B2*-*1* or *MaERF*-*B2*-*3*. To understand the changes in gene expression that takes place after flooding, the expression of the *MaERF*-*B2*-*1* and *MaERF*-*B2*-*2* was detected after treatment lasting 1 h, 3 h, 5 h, 7 h, 1 day, 3 days, 5 days, and 7 days (Fig. 6c). Results showed that *MaERF*-*B2*-*1* was up-regulated in roots and leaves after 1 day of flooding. The change took place faster in leaves than that in roots. *MaERFB2*-*2* showed fast up-regulation in roots after 1 h of flooding. *MaERF*-*B2*-*1* showed a greater fold change than *MaERF*-*B2*-*2*. These results indicated that *MaERF*-*B2*-*1* and *MaERFB2*-*2* may play important roles in Husang-32’s response to flooding.

**Table 2:**
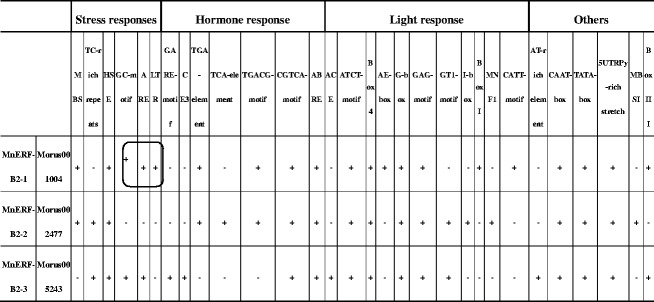
*Cis*-elements in *MnERF*-*B2*-*1*, *MnERF*-*B2*-*2*, and *MnERF*-*B2*-*3* promoters

## Discussion

Genes such as *ACS*, *ACO*, and *EIN3* are essential targets in the biosynthesis of ethylene and the transduction of this signal. In *Arabidopsis*, ACS is considered a rate-limiting synthase in ethylene biosynthesis (Jakubowicz and Sadowski 2002). The phosphorylation sites of ACS are important to rapid turnover during the response to various kinds of stimulation (Joo et al. 2008). Previous research indicated *CmACS*-*7* affects the development of the stamina in female flowers (Boualem et al. 2008). The expression of *MnACS5* was restricted to female flowers in mulberry plants, suggesting that MnACS5, rather than an ACC synthase, may play a different role. ACO genes are responsible for ethylene production. In the present study, the expression profiles of four putative *MnACOs* were analyzed and variation was observed in their levels of expression. Mulberry *ACO* genes, such as *MnACO1* and *MnACO2*, whose expression takes place mainly in fruit, might be involved in the regulation of ethylene during fruit ripening. In ethylene signal transduction, EIL and EIN3 proteins are crucial transcriptional factors. They are bound to the promoters of downstream genes. In this way, they regulate a wide range of stress responses (Chao et al. 1997). *MnEIN3*, *MnEIL1*, *MnEIL3*, and *MnEIL5* have no introns. *MnEIN3*, *MnEIL1*, *MnEIL4*, and *MnEIL5* form tandem arrays in the mulberry genome, and the genes in these two pairs are not only similar in sequence but in pattern of expression, suggesting the existence of complicated regulation affecting those processes.

The AP2/ERF family includes plant-specific transcription factors called ethylene response element binding proteins (EREBPs). EREBPs were originally identified through their ability to bind to the GCC box, a DNA motif associated with ethylene and pathogen-induced gene expression (Fujimoto et al. 2000). In the present study, 116 of AP2/ERF genes were identified in the mulberry genome. A phylogenetic analysis of AP2/ERF genes in mulberry plants and *Arabidopsis* showed mulberry plants to have more AP2/ERF genes in V group. Two research groups have shown that the overexpression of *WIN1*/*SHN1* (*At1g15360*), an *Arabidopsis* AP2/ERF gene in V group, resulted in the enhanced accumulation of epidermal wax (Aharoni et al. 2004; Broun et al. 2004). The expansion of mulberry AP2/ERF genes in V group is of particular interest.

Flooding is a serious matter in China’s Three Gorges Reservoir Region. It has a profound effect on mulberry growth and development. Previous studies on rice have shown that the genes at the *Submergence1* (*Sub1*) locus improve tolerance to submergence. This locus includes three ERF transcriptional factors (*Sub1A*, *Sub1B*, and *Sub1C*) (Jung et al. 2010). In *Arabidopsis*, the *RAP2.12* and *RAP2.2* genes became active in response to water-logging. In flooding, the expression of *RAP2.12* is up-regulated and the product accumulates in the nucleus to activate the expression of hypoxia genes (Licausi et al. 2011). The overexpression of *RAR2.2* improves plant survival under hypoxia stress conditions (Hinz et al. 2010). Three mulberry genes, *MaERF*-*B2*-*1*, *MaERF*-*B2*-*2*, and *MaERF*-*B2*-*3*, appear to be the counterparts of *RAP2.12*, *RAP2.2*, and *RAP2.3* based on the sequences. All of them contain conserved N-terminal sequences, which are important in response to hypoxia. Moreover, the presences of ARE and GC *cis*-elements in the promoters of *MnERF*-*B2*-*1* and *MnERF*-*B2*-*3* could lead to a proposal that these two genes might be involved in dealing with hypoxia. Our data further indicated that the patterns of expression of *MaERF*-*B2*-*1* and *MaERF*-*B2*-*2* changed in response to flooding treatment.

## Electronic supplementary material

Below is the link to the electronic supplementary material.

Supplementary Table 1 (DOCX 23 kb)

Supplementary Table 2 (DOCX 14 kb)

Supplemental figure 1 Alignment of amino acid sequences of five *MnACS* genes using the ClustalX program. Conserved residues are shaded in black. The 11 black circles designate the residues that represent the conserved amino acids in aminotransferases. The conserved glutamate residue (E) is marked with an grey circle. Three black arrows indicate the Val and Ser residues which are the sites for phosphorylation. The Ser residues that are targets of the MPK6 kinase are marked with asterisks. (JPEG 5318 kb)

Supplemental figure 2 Alignment of amino acid sequences of *MnACO* genes. Fe (II)-binding motif and two cosubstrate-binding motifs are marked with red dash box, and red boxes, respectively. The triangles designate the sites of the cysteines. The other important amino acid are marked with the asterisks. (JPEG 4846 kb)

Supplemental figure 3 Alignment of amino acid sequences of *MnETR* genes. Regions I, II, and III represent three transmembrane fragments. (JPEG 6306 kb)

Supplemental figure 4 Alignment of amino acid sequences of *MnEIN3* genes. The amino-terminal acidic domain (AD), pro-rich region (PR), and five small basic domain (BDI-V) are labeled. The poly-Gln and Poly-Asn repeats at the C-terminal portion are marked with triangles. The arrow indicates the Lys residue essential for the function of EIN3. (JPEG 4085 kb)

Supplemental figure 5 Phylogenetic tree of the predicted mulberry AP2/ERF proteins. A phylogenetic tree of AP2/ERF was constructed using maximum-likelihood method in PhyML 3.0 with a Perl script. 15 groups are marked. (JPEG 3240 kb)

Supplemental figure 6 Exon/intron structures of mulberry AP2/ERF genes. Different exon/intron structures of genes were plotted using the GSDS server. The intron phase indicates the position of the intron within a codon. If the codon is not located within a codon or is located between two codons, the phase is 0. Phase 2 designates introns between the first and second bases of a codon and phase 2 designates introns between the second and third bases of a codon. (JPEG 1571 kb)

## References

[CR1] Abeles FB, Morgan PW, Saltveit ME (1992). Ethylene in plant biology.

[CR2] Adams D, Yang S (1979). Ethylene biosynthesis: identification of 1-aminocyclopropane-1-carboxylic acid as an intermediate in the conversion of methionine to ethylene. Proc Natl Acad Sci U S A.

[CR3] Adams-Phillips L, Barry C, Giovannoni J (2004). Signal transduction systems regulating fruit ripening. Trends Plant Sci.

[CR4] Aharoni A, Dixit S, Jetter R, Thoenes E, van Arkel G, Pereira A (2004). The SHINE clade of AP2 domain transcription factors activates wax biosynthesis, alters cuticle properties, and confers drought tolerance when overexpressed in *Arabidopsis*. Plant Cell.

[CR5] Alonso JM, Hirayama T, Roman G, Nourizadeh S, Ecker JR (1999). *EIN2*, a bifunctional transducer of ethylene and stress responses in *Arabidopsis*. Science.

[CR6] Blumenthal S, Hendrickson H, Abrol Y, Conn EE (1968). Cyanide metabolism in higher plants III. The biosynthesis of β-cyanoalanine. J Biol Chem.

[CR7] Boualem A, Fergany M, Fernandez R, Troadec C, Martin A, Morin H (2008). A conserved mutation in an ethylene biosynthesis enzyme leads to andromonecy in melons. Science.

[CR8] Broun P, Poindexter P, Osborne E, Jiang CZ, Riechmann JL (2004). *WIN1*, a transcriptional activator of epidermal wax accumulation in *Arabidopsis*. Proc Natl Acad Sci U S A.

[CR9] Chao Q, Rothenberg M, Solano R, Roman G, Terzaghi W (1997). Activation of the ethylene gas response pathway in *Arabidopsis* by the nuclear protein ETHYLENE-INSENSITIVE3 and related proteins. Cell.

[CR10] Dietz KJ, Vogel MO, Viehhauser A (2010). AP2/EREBP transcription factors are part of gene regulatory networks and integrate metabolic, hormonal and environmental signals in stress acclimation and retrograde signalling. Protoplasma.

[CR11] Fujimoto SY, Ohta M, Usui A, Shinshi H, Ohme-Takagi M (2000). *Arabidopsis* ethylene-responsive element binding factors act as transcriptional activators or repressors of GCC box-mediated gene expression. Plant Cell.

[CR12] Gao Z, Chen YF, Randlett MD, Zhao XC, Findell JL, Kieber JJ, Schaller GE (2003). Localization of the Raf-like kinase *CTR1* to the endoplasmic reticulum of *Arabidopsis* through participation in ethylene receptor signaling complexes. J Biol Chem.

[CR13] Guo AY, Zhu QH, Chen X, Luo JC (2007). GSDS: a gene structure display server. Yi Chuan.

[CR14] Hinz M, Wilson IW, Yang J, Buerstenbinder K, Llewellyn D, Dennis ES, Sauter M, Dolferus R (2010). *Arabidopsis RAP2.2*: an ethylene response transcription factor that is important for hypoxia survival. Plant Physiol.

[CR15] Jakubowicz M, Sadowski J (2002). 1-aminocyclopropane-1-carboxylate synthase—genes and expression. Acta Physiol Plant.

[CR16] Johnson PR, Ecker JR (1998). The ethylene gas signal transduction pathway: a molecular perspective. Annu Rev Genet.

[CR17] Joo S, Yd L, Lueth A, Zhang S (2008). MAPK phosphorylation-induced stabilization of ACS6 protein is mediated by the non-catalytic C-terminal domain, which also contains the cis-determinant for rapid degradation by the 26S proteasome pathway. Plant J.

[CR18] Jung KH, Seo YS, Walia H, Cao P, Fukao T, Canlas PE, Amonpant F, Bailey-Serres J, Ronald PC (2010). The submergence tolerance regulator *Sub1A* mediates stress-responsive expression of AP2/ERF transcription factors. Plant Physiol.

[CR19] Kagale S, Links MG, Rozwadowski K (2010). Genome-wide analysis of ethylene-responsive element binding factor-associated amphiphilic repression motif-containing transcriptional regulators in *Arabidopsis*. Plant Physiol.

[CR20] Larkin M, Blackshields G, Brown N, Chenna R, McGettigan PA, McWilliam H, Valentin F, Wallace IM, Wilm A, Lopez R (2007). Clustal W and clustal X version 2.0. Bioinformatics.

[CR21] Lescot M, Déhais P, Thijs G, Marchal K, Moreau Y, Van de Peer Y, Rouzé P, Rombauts S (2002). PlantCARE, a database of plant cis-acting regulatory elements and a portal to tools for in silico analysis of promoter sequences. Nucleic Acids Res.

[CR22] Letunic I, Doerks T, Bork P (2012). SMART 7: recent updates to the protein domain annotation resource. Nucleic Acids Res.

[CR23] Licausi F, Kosmacz M, Weits DA, Giuntoli B, Giorgi FM, Voesenek LA, Perata P, van Dongen JT (2011). Oxygen sensing in plants is mediated by an N-end rule pathway for protein destabilization. Nature.

[CR24] Lin Z, Zhong S, Grierson D (2009). Recent advances in ethylene research. J Exp Bot.

[CR25] Miyazaki JH, Yang SF (1987). The methionine salvage pathway in relation to ethylene and polyamine biosynthesis. Physiol Plant.

[CR26] Mortazavi A, Williams BA, McCue K, Schaeffer L, Wold B (2008). Mapping and quantifying mammalian transcriptomes by RNA-Seq. Nat Methods.

[CR27] Ohta M, Matsui K, Hiratsu K, Shinshi H, Ohme-Takagi M (2001). Repression domains of class II ERF transcriptional repressors share an essential motif for active repression. Plant Cell.

[CR28] Rodrıguez FI, Esch JJ, Hall AE, Binder BM, Schaller GE, Bleecker AB (1999). A copper cofactor for the ethylene receptor *ETR1* from *Arabidopsis*. Science.

[CR29] Voesenek L, Blom C (1989). Growth responses of Rumex species in relation to submergence and ethylene. Plant Cell Environ.

[CR30] Yang SF, Hoffman NE (1984). Ethylene biosynthesis and its regulation in higher plants. Annu Rev Plant Physiol.

[CR31] Zhang L, Li Z, Quan R, Li G, Wang R, Huang R (2011). An AP2 domain-containing gene, *ESE1*, targeted by the ethylene signaling component *EIN3* is important for the salt response in *Arabidopsis*. Plant Physiol.

[CR32] Zhong S, Shi H, Xue C, Wang L, Xi YP, Li JG, Quail PH, Deng XW, Guo HW (2012). A molecular framework of light-controlled phytohormone action in *Arabidopsis*. Curr Biol.

