# Supplementary material for: Identification of the mulberry genes involved in ethylene biosynthesis and signaling pathways and the expression of MaERF-B2-1 and MaERF-B2-2 in the response to flooding stress
Source: Funct Integr Genomics. 2014 Sep 18;14(4):767–77. doi: 10.1007/s10142-014-0403-2 (PMC4233114; doi:10.1007/s10142-014-0403-2)
Supplement: Supplementary file 1 — (DOCX 23 kb) [file 10142_2014_403_MOESM1_ESM.docx]

**Supplemental Table 1. AP2/ERF genes in the *M. notabilis* genome.**

| **Classification** | **Genes** | **Accession NO** | **CDS length** | **Exons** | **Scaffold (strand):start-end** |
| --- | --- | --- | --- | --- | --- |
| AP2 family | MnAP2-1 | Morus000912 | 249 | 1 | scaffold7344:19421-19669:- |
|  | MnAP2-2 | Morus001314 | 1782 | 8 | scaffold959:3169-7118:- |
|  | MnAP2-3 | Morus001747 | 1560 | 10 | scaffold2193:17746-21057:- |
|  | MnAP2-4 | Morus001950 | 1200 | 7 | scaffold2080:48316-50518:- |
|  | MnAP2-5 | Morus003848 | 1338 | 10 | scaffold845:4745-7544:- |
|  | MnAP2-6 | Morus004404 | 1065 | 7 | scaffold1527:20107-23748:+ |
|  | MnAP2-7 | Morus005589 | 2193 | 8 | scaffold2693:112497-116313:- |
|  | MnAP2-8 | Morus005779 | 1545 | 10 | scaffold312:156481-159406:- |
|  | MnAP2-9 | Morus007080 | 2157 | 8 | scaffold991:12065-15520:+ |
|  | MnAP2-10 | Morus008563 | 1176 | 7 | scaffold966:87597-90248:+ |
|  | MnAP2-11 | Morus011196 | 1944 | 12 | scaffold160:142805-151348:+ |
|  | MnAP2-12 | Morus012990 | 2010 | 8 | scaffold679:88476-91766:- |
|  | MnAP2-13 | Morus015259 | 1722 | 8 | scaffold1005:4104-6976:+ |
|  | MnAP2-14 | Morus015984 | 1356 | 8 | scaffold174:315594-320368:- |
|  | MnAP2-15 | Morus016770 | 1152 | 6 | scaffold499:520269-523629:- |
|  | MnAP2-16 | Morus018417 | 402 | 1 | scaffold89:267857-268258:+ |
|  | MnAP2-17 | Morus020261 | 519 | 4 | scaffold1333:632129-633738:+ |
|  | MnAP2-18 | Morus021093 | 1239 | 7 | scaffold155:409782-412211:+ |
|  | MnAP2-19 | Morus026782 | 1689 | 9 | scaffold184:623515-628778:+ |
|  | MnAP2-20 | Morus028103 | 1137 | 7 | scaffold78:3446205-3448429:- |
|  | MnAP2-21 | Morus028104 | 1887 | 7 | scaffold78:3450076-3456448:- |
| MnDREB-A1 | MnDREB-A1-1 | Morus001049 | 720 | 1 | scaffold2527:30142-30861:- |
|  | MnDREB-A1-2 | Morus013794 | 762 | 1 | scaffold705:206444-207205:+ |
| MnDREB-A2 | MnDREB-A2-1 | Morus002388 | 858 | 1 | scaffold1514:71420-72277:+ |
|  | MnDREB-A2-2 | Morus008736 | 1209 | 2 | scaffold635:205485-209307:- |
|  | MnDREB-A2-3 | Morus009488 | 2232 | 5 | scaffold258:179200-222235:- |
|  | MnDREB-A2-4 | Morus012269 | 1101 | 1 | scaffold65:815-1915:- |
|  | MnDREB-A2-5 | Morus019405 | 2349 | 11 | scaffold262:175528-181701:+ |
|  | MnDREB-A2-6 | Morus024469 | 600 | 1 | scaffold205:96374-96973:- |
| MnDREB-A3 | MnDREB-A3-1 | Morus009903 | 1077 | 1 | scaffold1198:218162-219238:+ |
| MnDREB-A4 | MnDREB-A4-1 | Morus001767 | 786 | 1 | scaffold2017:19329-20114:- |
|  | MnDREB-A4-2 | Morus010702 | 750 | 1 | scaffold245:67210-67959:+ |
|  | MnDREB-A4-3 | Morus010902 | 804 | 1 | scaffold2235:288837-289640:- |
|  | MnDREB-A4-4 | Morus013800 | 639 | 1 | scaffold705:244778-245416:- |
|  | MnDREB-A4-5 | Morus015729 | 630 | 2 | scaffold759:28555-29350:- |
|  | MnDREB-A4-6 | Morus021807 | 771 | 2 | scaffold1009:332505-333348:- |
|  | MnDREB-A4-7 | Morus023492 | 627 | 1 | scaffold404:772131-772757:- |
|  | MnDREB-A4-8 | Morus024074 | 816 | 1 | scaffold789:559343-560158:+ |
|  | MnDREB-A4-9 | Morus026252 | 804 | 1 | scaffold307:635582-636385:+ |
| MnDREB-A5 | MnDREB-A5-1 | Morus002530 | 636 | 1 | scaffold260:21623-22258:+ |
|  | MnDREB-A5-2 | Morus002531 | 516 | 1 | scaffold260:28623-29138:+ |
|  | MnDREB-A5-3 | Morus003928 | 567 | 1 | scaffold1387:80555-81121:- |
|  | MnDREB-A5-4 | Morus007221 | 534 | 1 | scaffold1139:39314-39847:- |
|  | MnDREB-A5-5 | Morus008873 | 540 | 1 | scaffold523:50835-51374:- |
|  | MnDREB-A5-6 | Morus014282 | 477 | 1 | scaffold396:28673-29149:+ |
|  | MnDREB-A5-7 | Morus018629 | 843 | 1 | scaffold348:459349-460191:+ |
|  | MnDREB-A5-8 | Morus020177 | 777 | 1 | scaffold570:473347-474123:- |
|  | MnDREB-A5-9 | Morus025497 | 633 | 1 | scaffold485:412920-413552:+ |
| MnDREB-A6 | MnDREB-A6-1 | Morus000151 | 963 | 1 | C11421298:504-1466:+ |
|  | MnDREB-A6-2 | Morus003883 | 963 | 1 | scaffold540:70586-71548:- |
|  | MnDREB-A6-3 | Morus003964 | 1008 | 1 | scaffold2021:130905-131912:- |
|  | MnDREB-A6-4 | Morus011587 | 1323 | 1 | scaffold732:302337-303659:- |
|  | MnDREB-A6-5 | Morus021553 | 1065 | 1 | scaffold206:509562-510626:+ |
|  | MnDREB-A6-6 | Morus021618 | 1173 | 1 | scaffold783:443007-444179:- |
| MnERF-B1 | MnERF-B1-1 | Morus005756 | 675 | 1 | scaffold1229:134344-135018:+ |
|  | MnERF-B1-2 | Morus007569 | 969 | 1 | scaffold362:123540-124508:+ |
|  | MnERF-B1-3 | Morus009827 | 636 | 1 | scaffold832:174582-175217:+ |
|  | MnERF-B1-4 | Morus010314 | 687 | 1 | scaffold290:70313-70999:+ |
|  | MnERF-B1-5 | Morus012460 | 726 | 1 | scaffold1436:143242-143967:- |
|  | MnERF-B1-6 | Morus015072 | 708 | 1 | scaffold744:375582-376289:+ |
|  | MnERF-B1-7 | Morus015075 | 552 | 1 | scaffold744:396153-396704:- |
|  | MnERF-B1-8 | Morus023587 | 1089 | 1 | scaffold156:870241-871329:+ |
|  | MnERF-B1-9 | Morus025687 | 849 | 1 | scaffold843:189739-190587:+ |
|  | MnERF-B1-10 | Morus027718 | 1344 | 1 | scaffold99:1114983-1116326:+ |
| MnERF-B2 | MnERF-B2-1 | Morus001004 | 828 | 2 | scaffold3575:13661-14587:+ |
|  | MnERF-B2-2 | Morus002477 | 1167 | 2 | scaffold2802:24185-26596:- |
|  | MnERF-B2-3 | Morus005243 | 825 | 2 | scaffold824:93705-95271:- |
| MnERF-B3 | MnERF-B3-1 | Morus000238 | 900 | 1 | Scaffold10079:534-1433:+ |
|  | MnERF-B3-2 | Morus001232 | 636 | 1 | scaffold4939:2231-2866:+ |
|  | MnERF-B3-3 | Morus001662 | 1224 | 4 | scaffold1496:8834-13133:+ |
|  | MnERF-B3-4 | Morus001663 | 603 | 1 | scaffold1496:17938-18540:- |
|  | MnERF-B3-5 | Morus009794 | 408 | 1 | scaffold40:198424-198831:+ |
|  | MnERF-B3-6 | Morus009795 | 483 | 1 | scaffold40:209340-209822:- |
|  | MnERF-B3-7 | Morus009796 | 678 | 1 | scaffold40:224225-224902:+ |
|  | MnERF-B3-8 | Morus009797 | 783 | 1 | scaffold40:228418-229200:+ |
|  | MnERF-B3-9 | Morus009798 | 870 | 1 | scaffold40:232108-232977:- |
|  | MnERF-B3-10 | Morus012823 | 438 | 1 | scaffold147:220670-221107:+ |
|  | MnERF-B3-11 | Morus012825 | 531 | 1 | scaffold147:288345-288875:- |
|  | MnERF-B3-12 | Morus012827 | 651 | 1 | scaffold147:376893-377543:+ |
|  | MnERF-B3-13 | Morus017066 | 828 | 1 | scaffold223:46312-47139:- |
|  | MnERF-B3-14 | Morus021847 | 528 | 1 | scaffold1009:583179-583706:- |
|  | MnERF-B3-15 | Morus021848 | 783 | 1 | scaffold1009:586656-587438:- |
|  | MnERF-B3-16 | Morus021849 | 375 | 2 | scaffold1009:589443-589893:- |
|  | MnERF-B3-17 | Morus021850 | 765 | 1 | scaffold1009:597102-597866:- |
|  | MnERF-B3-18 | Morus021851 | 588 | 1 | scaffold1009:606694-607281:+ |
|  | MnERF-B3-19 | Morus024015 | 1050 | 1 | scaffold789:28056-29105:- |
|  | MnERF-B3-20 | Morus024017 | 870 | 1 | scaffold789:51462-52331:+ |
|  | MnERF-B3-21 | Morus024526 | 870 | 1 | scaffold205:526715-527584:- |
| MnERF-B4 | MnERF-B4-1 | Morus003975 | 783 | 2 | scaffold2518:94266-95707:+ |
|  | MnERF-B4-2 | Morus003977 | 801 | 2 | scaffold2518:118614-120073:+ |
|  | MnERF-B4-3 | Morus004476 | 1455 | 2 | scaffold1695:150104-152057:+ |
|  | MnERF-B4-4 | Morus006711 | 1008 | 2 | scaffold84:26956-28945:+ |
|  | MnERF-B4-5 | Morus013140 | 816 | 2 | scaffold324:69126-71944:- |
| MnERF-B5 | MnERF-B5-1 | Morus003067 | 885 | 1 | scaffold1016:46107-46991:+ |
|  | MnERF-B5-2 | Morus011468 | 1107 | 1 | scaffold812:218098-219204:- |
|  | MnERF-B5-3 | Morus012079 | 1002 | 1 | scaffold118:265041-266042:- |
|  | MnERF-B5-4 | Morus018807 | 738 | 1 | scaffold227:499526-500263:- |
| MnERF-B6 | MnERF-B6-1 | Morus003703 | 870 | 1 | scaffold1658:55641-56510:+ |
|  | MnERF-B6-2 | Morus009627 | 930 | 1 | scaffold1404:52649-53578:+ |
|  | MnERF-B6-3 | Morus011629 | 822 | 2 | scaffold453:14243-15471:+ |
|  | MnERF-B6-4 | Morus011824 | 1119 | 1 | scaffold448:278922-280040:- |
|  | MnERF-B6-5 | Morus014457 | 612 | 2 | scaffold250:252764-253474:+ |
|  | MnERF-B6-6 | Morus019090 | 843 | 1 | scaffold176:93917-94759:- |
|  | MnERF-B6-7 | Morus020947 | 1248 | 1 | scaffold241:3853-5100:+ |
|  | MnERF-B6-8 | Morus022246 | 687 | 1 | scaffold16:98149-98835:+ |
|  | MnERF-B6-9 | Morus023696 | 543 | 1 | scaffold436:865601-866143:+ |
|  | MnERF-B6-10 | Morus025682 | 588 | 2 | scaffold843:72528-73214:+ |
|  | MnERF-B6-11 | Morus025785 | 786 | 2 | scaffold843:1054635-1056254:+ |
|  | MnERF-B6-12 | Morus014665 | 1023 | 1 | scaffold6:288693-289715:- |
|  | MnERF-B6-13 | Morus019973 | 1026 | 1 | scaffold2035:470168-471193:- |
|  | MnERF-B6-14 | Morus002710 | 732 | 1 | scaffold292:65989-66720:- |
|  | MnERF-B6-15 | Morus027531 | 951 | 1 | scaffold45:1318392-1319342:+ |
| MnRAV | MnRAV-1 | Morus009976 | 1191 | 2 | scaffold360:33725-35557:+ |
|  | MnRAV-2 | Morus018415 | 1149 | 1 | scaffold89:252157-253305:+ |
|  | MnRAV-3 | Morus022972 | 1137 | 1 | scaffold538:749582-750718:+ |
| Soloist | Soloist | Morus007011 | 714 | 6 | scaffold158:80188-82782:+ |
|  |  |  |  |  |  |
