# Supplementary material for: Identification of the mulberry genes involved in ethylene biosynthesis and signaling pathways and the expression of MaERF-B2-1 and MaERF-B2-2 in the response to flooding stress
Source: Funct Integr Genomics. 2014 Sep 18;14(4):767–77. doi: 10.1007/s10142-014-0403-2 (PMC4233114; doi:10.1007/s10142-014-0403-2)
Supplement: Supplementary file 2 — (DOCX 14 kb) [file 10142_2014_403_MOESM2_ESM.docx]

| **Supplement Table 2. Primers used for PCR and RT-qPCR verification** | | |
| --- | --- | --- |
| **Gene** | **Forward primer(5’-3’)** | **Reverse primer(5’-3’)** |
| qMnACS1 | TCCAATGTTCCGAGCCTG | TCCCGACGAAAGACCTAA |
| qMnACS2 | CCTTGGAAGAAGCGTATGAG | GTGGCTGCGTAGATTTCG |
| qMnACS3 | ACAAGTTCTCCGAAGTCT | GTCTGCGAGGATACCAAA |
| qMnACS4 | GAATGTGCTTCGCCAATA | GTCAGTTTCACGATGGTC |
| qMnACS5 | AGAGGCGTTCTCATCACA | CGACCCCGAGTAGATTTC |
| qMnACO1 | CCCTCTGTGGGAACTAA | TTGGAGACCTGGGACTTG |
| qMnACO2 | TAATGTCCCCCCAATGCG | ATCCTGTTACCGTCCGTT |
| qMnACO3 | TCCTGGACCTGTTGTGTGA | ATGGTGGGTAGTTGCTGAC |
| qMnACO4 | TTCCTTCTACAATCCCTC | GAACTTCTGGTCAGCATA |
| qMnETR1 | ATGGGGTAAGCAGGTCAG | AGTTCGTAACCATCCGTG |
| qMnETR2 | GGAAGTTTAGGAGCCGTAG | CATACACCTATCCCAGACAT |
| qMnERS1 | CACCACCACTGTTACTCC | TGGTAATGCGGTGAAGTT |
| qMnEIN4 | GGGATGTGGAGACAGAGT | CTCCTTAACTTCGCCGCT |
| qMnEIN3 | GTATTGCTGGTCTCGGTCTT | TGCTCGGTAGCCGTAAAA |
| qMnEIL1 | GATTACCGTCTTGGCTTTC | GCTGAGGACCTGGCTTA |
| qMnEIL3 | GGCTTAGGTTCTGTTTCA | TGTTATTCCGCTGTTTGC |
| qMnEIL4 | GCCTCACAATCTCATCAAC | CACAAAGCCGAGTCCTAT |
| qMnEIL5 | TTGGTCGGACAGTCATTG | TTGGTGAGTTGGGAAAGG |
| qMaERF-B2-1 | AAGGGGTTCGTGTTTGGCTC | CCGTAAGTGTCGTTGGTGTCC |
| qMaERF-B2-2 | AGTGTTCCGACTTTGGGTTGG | GCTTCTTTGTTGGGTTAGTATCCTC |
| MaERF-B2-1 | CGTCCAAGTCCAACAAGC | ACCCCGACGAAGGAACAT |
| MaERF-B2-2 | GACGAATCCGTGGTAAGA | GTGTCCAATAACGCATCC |
| kl-MaERF-B2-1 | ATGTGTGGCGGTGCTGTAAT | TTACACATCGGACCCCGAG |
| kl-MaERF-B2-2 | ATGTGTGGAGGTGCTATAATCT | TTAGAAGGCTTCGCCAGACA |
