# Supplementary figures and images for: Identification of the mulberry genes involved in ethylene biosynthesis and signaling pathways and the expression of MaERF-B2-1 and MaERF-B2-2 in the response to flooding stress
Source: Funct Integr Genomics. 2014 Sep 18;14(4):767–77. doi: 10.1007/s10142-014-0403-2 (PMC4233114; doi:10.1007/s10142-014-0403-2)

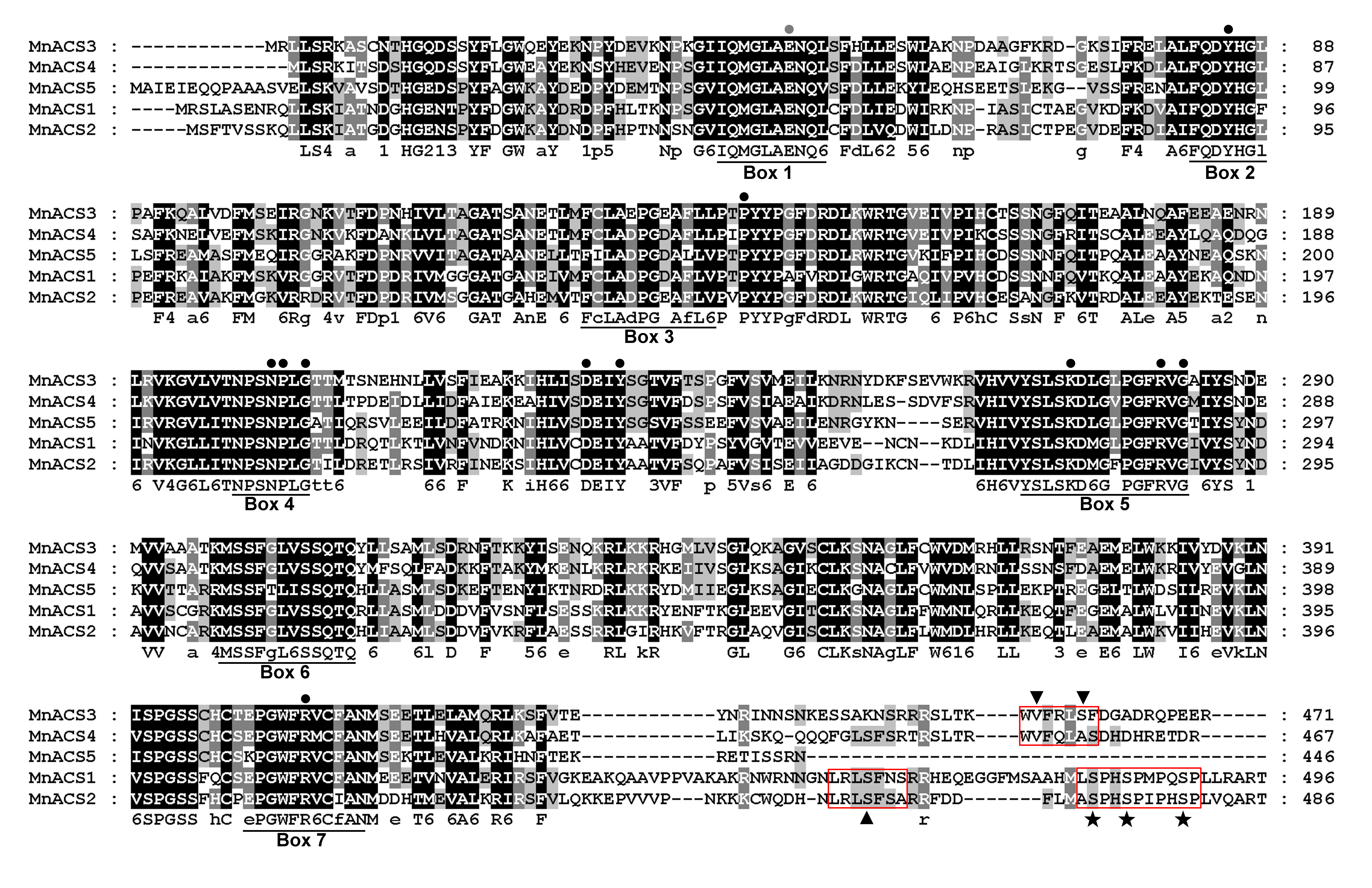

Supplement: Supplementary file 3 — Alignment of amino acid sequences of five MnACS genes using the ClustalX program. Conserved residues are shaded in black. The 11 black circles designate the residues that represent the conserved amino acids in aminotransferases. The conserved glutamate residue (E) is marked with an grey circle. Three black arrows indicate the Val and Ser residues which are the sites for phosphorylation. The Ser residues that are targets of the MPK6 kinase are marked with asterisks. (JPEG 5318 kb) [file 10142_2014_403_Fig7_ESM.jpg]

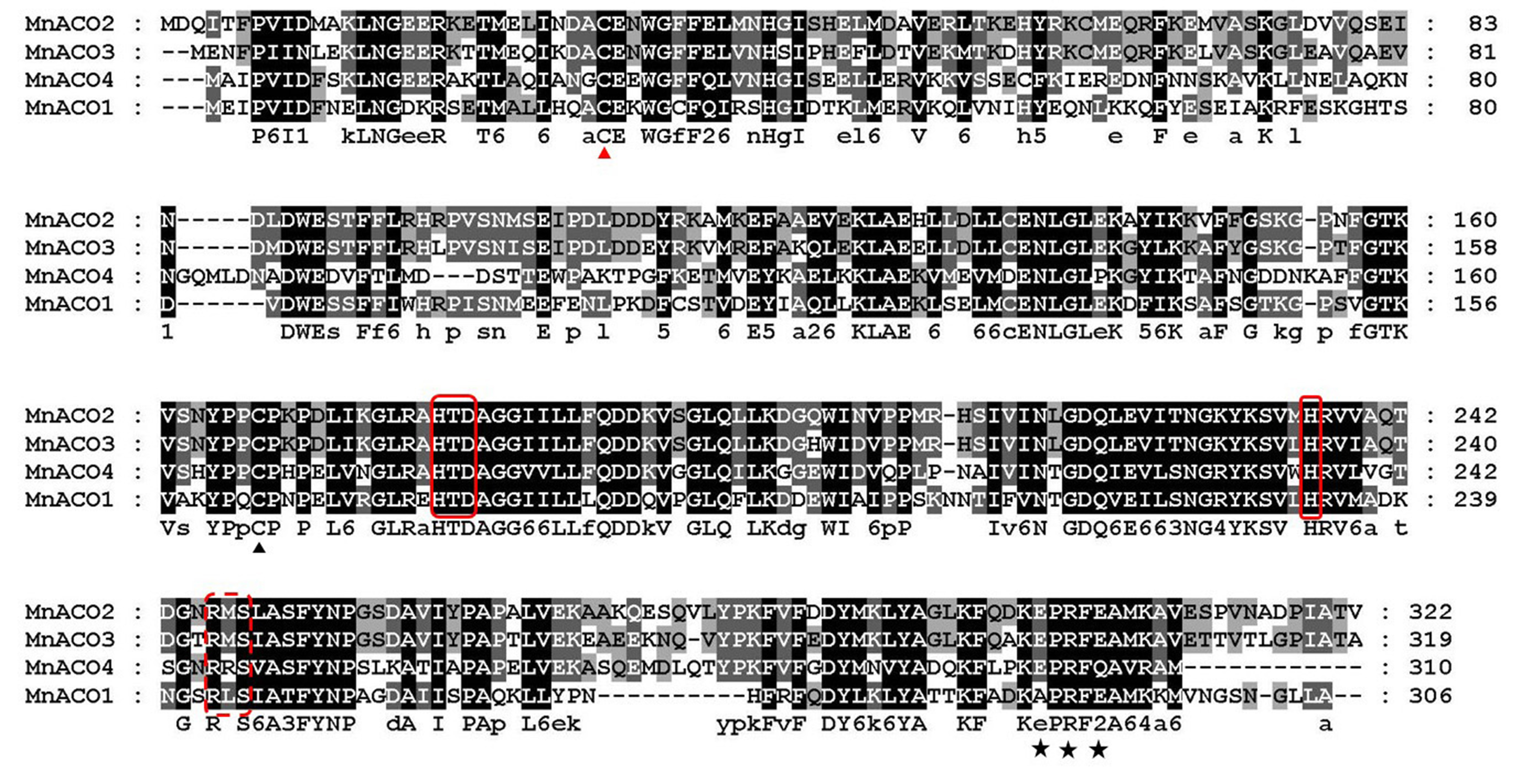

Supplement: Supplementary file 4 — Alignment of amino acid sequences of MnACO genes. Fe (II)-binding motif and two cosubstrate-binding motifs are marked with red dash box, and red boxes, respectively. The triangles designate the sites of the cysteines. The other important amino acid are marked with the asterisks. (JPEG 4846 kb) [file 10142_2014_403_Fig8_ESM.jpg]

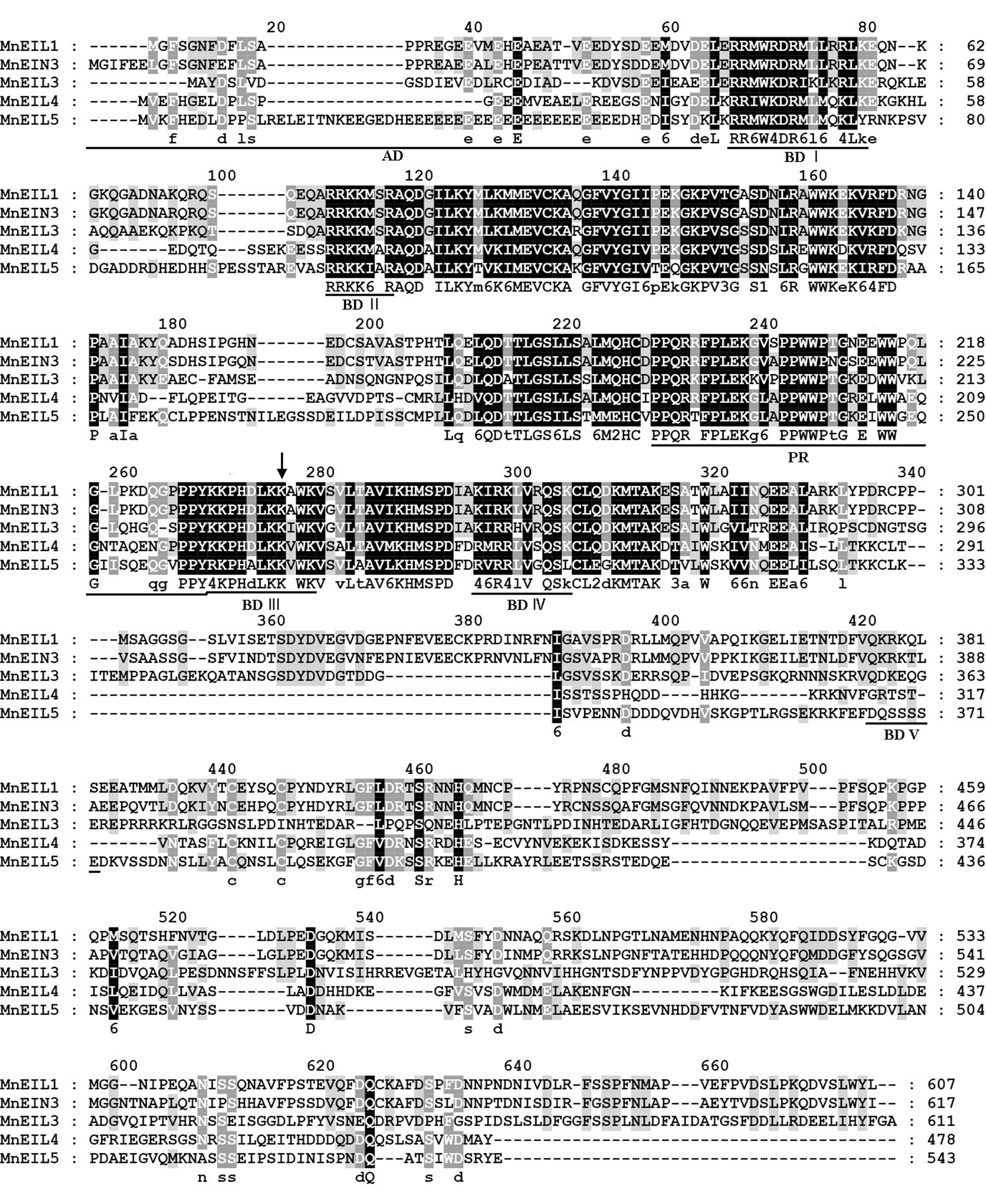

Supplement: Supplementary file 6 — Alignment of amino acid sequences of MnEIN3 genes. The amino-terminal acidic domain (AD), pro-rich region (PR), and five small basic domain (BDI-V) are labeled. The poly-Gln and Poly-Asn repeats at the C-terminal portion are marked with triangles. The arrow indicates the Lys residue essential for the function of EIN3. (JPEG 4085 kb) [file 10142_2014_403_Fig10_ESM.jpg]

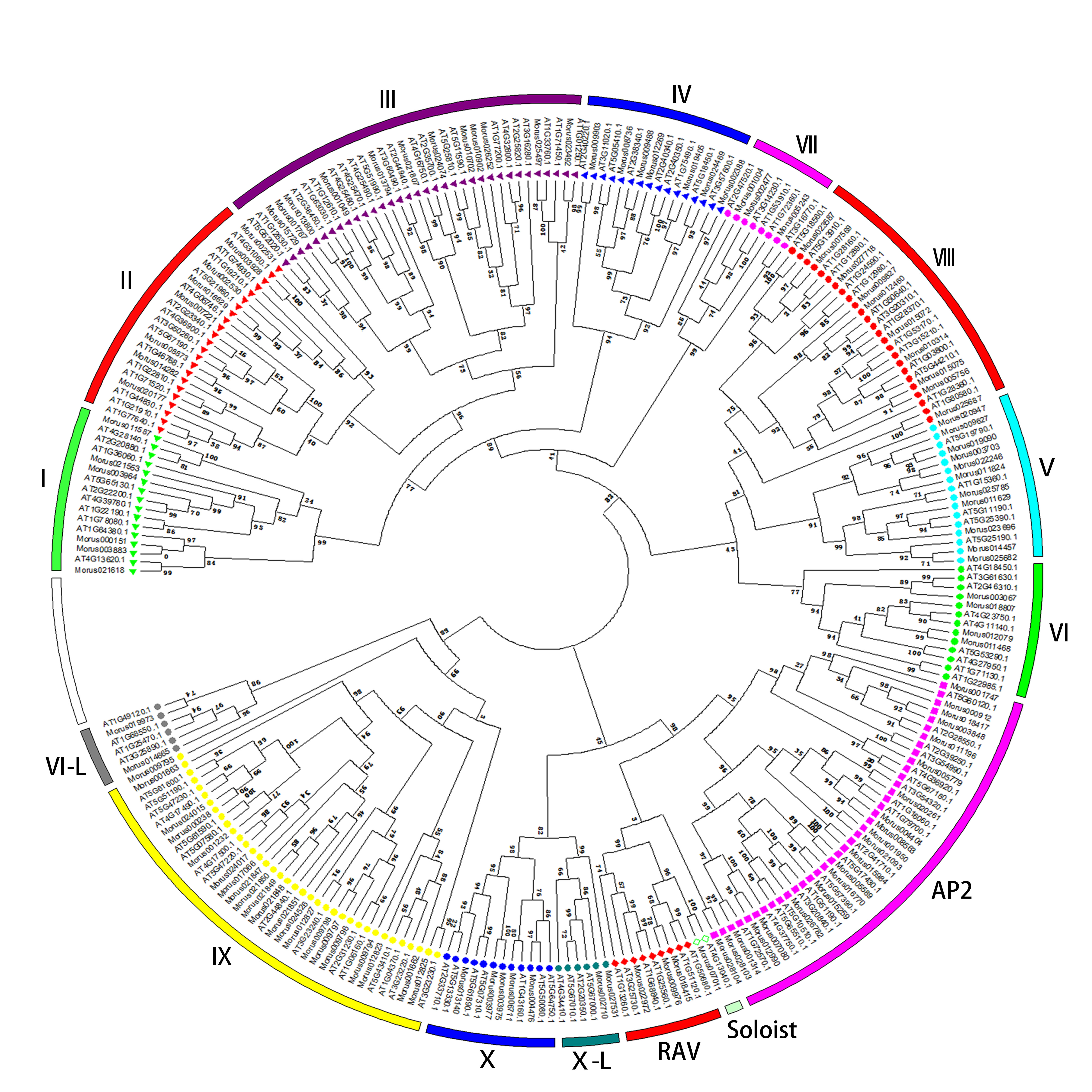

Supplement: Supplementary file 7 — Phylogenetic tree of the predicted mulberry AP2/ERF proteins. A phylogenetic tree of AP2/ERF was constructed using maximum-likelihood method in PhyML 3.0 with a Perl script. 15 groups are marked. (JPEG 3240 kb) [file 10142_2014_403_Fig11_ESM.jpg]

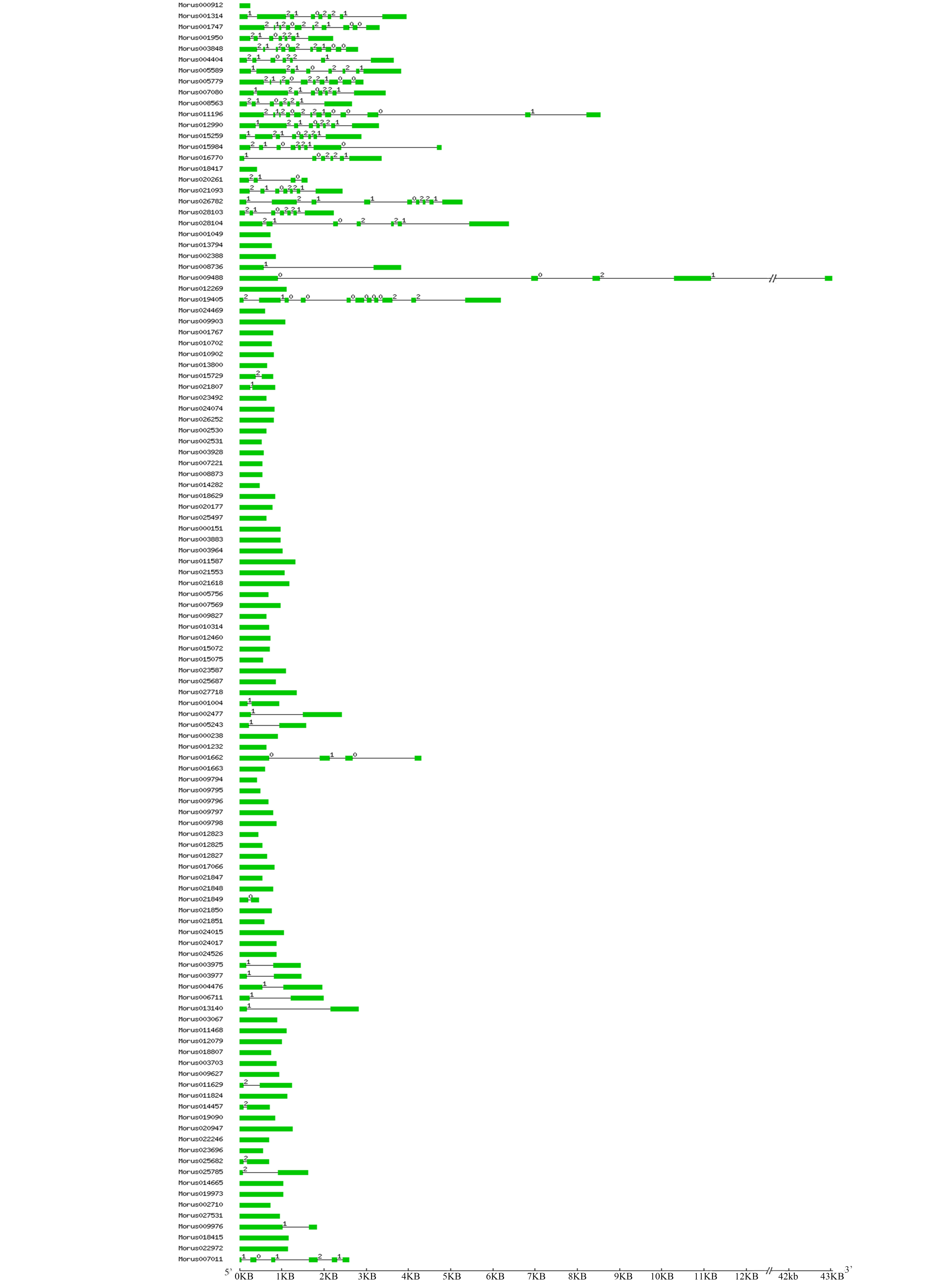

Supplement: Supplementary file 8 — Exon/intron structures of mulberry AP2/ERF genes. Different exon/intron structures of genes were plotted using the GSDS server. The intron phase indicates the position of the intron within a codon. If the codon is not located within a codon or is located between two codons, the phase is 0. Phase 2 designates introns between the first and second bases of a codon and phase 2 designates introns between the second and third bases of a codon. (JPEG 1571 kb) [file 10142_2014_403_Fig12_ESM.jpg]
